# Genetic variant rs7820258 regulates the expression of indoleamine 2,3-dioxygenase 1 in brain regions

**DOI:** 10.1073/pnas.2007022117

**Published:** 2020-09-29

**Authors:** Zhijie Han, Dian He, Yang Zhang

**Affiliations:** ^a^Department of Bioinformatics, School of Basic Medicine, Chongqing Medical University, Chongqing 400016, China;; ^b^Innovative Drug Research and Bioinformatics Group, School of Pharmaceutical Sciences, Chongqing University, Chongqing 401331, China;; ^c^Institute of Medicinal Chemistry, School of Pharmacy, Lanzhou University, Lanzhou 730000, China

Indoleamine 2,3-dioxygenase 1 (IDO1), the enzyme catalyzing the rate-limiting step along the kynurenine pathway, is widely known for being a powerful immune regulator via depletion of tryptophan, an essential amino acid, and production of kynurenines, some of which are endowed with immunoregulatory effects. In humans, IDO1 expression and activity are known to exhibit relatively large interindividual variability, particularly under pathological conditions, often as a result of single-nucleotide polymorphisms (SNPs) in the enzyme gene. Among these, IDO1 SNP rs7820268 was recently found to display a significantly different allele and genotype distribution among pediatric patients with autoimmune diabetes (type 1 diabetes [T1D]) and control subjects (1). Moreover, an association analysis test of rs7820268 genotypes using a genetic dominant model (CC versus T carriers) confirmed a significant association of the CC genotype with an increased risk of T1D. A significant reduction in IDO1 protein expression and levels of kynurenine (the main IDO1 product) but not transcripts was found to occur in association with the CC and CT but not TT genotype at rs7820268. Interestingly, restoration of IDO1 activity could be obtained by tocilizumab, an anti–IL-6 receptor antibody, in one-third of T1D patients mostly characterized by the CT genotype. Very recently, a significant and differential allele and genotype distribution was detected for the same IDO1 SNP in patients with relapsing-remitting multiple sclerosis (RRMS) compared to control subjects (2).

Evidence shows that analysis of expression quantitative trait loci (eQTLs) is an effective tool applied to identify the relationship between SNPs and gene expression (3, 4). Here, we selected four large-scale eQTLs datasets, including 2,116 (BIOS QTL Browser) (5), 5,311 (Blood eQTL browser) (6), 5,257 samples (FHS_eQTL) (7) in blood, and 134 in brain (Braineac) (8) from healthy individuals. Braineac provided the rs7820268 genotype and IDO1 expression data in 10 brain regions, which were subject to evaluation of their association using a linear regression analysis with age and gender as the covariance parameters by the R package “Matrix eQTL” (9). The other datasets offered the eQTLs results about the influences of rs7820268 on IDO1 expression. The threshold of significance was defined as *P* < 0.05.

Interestingly, we found that rs7820268 significantly affects IDO1 expression in blood and six brain tissues (*P* < 0.05). Particularly, rs7820268 T allele considerably up-regulates IDO1 expression in blood based on the three eQTLs datasets (β > 0), which is consistent with previous studies, namely, CC genotype at rs7820268 associating with deficient IDO1 activity in T1D and RRMS peripheral blood mononuclear cells. Similar results were observed in brain putamen (β = 0.21) and occipital cortex (β = 0.13). Besides, we found that rs7820268 T allele remarkably down-regulates IDO1 expression in brain cerebellar cortex (β = −0.12), substantia nigra (β = −0.14), thalamus (β = −0.13), and hippocampus (β = −0.18) (Table 1).

**Table 1.** Polymorphism rs7820268 T allele significantly affects IDO1 expression in brain and blood

Gene	SNP	Effect allele	*P* value	Effect size (β)	Tissues	Sample size	Dataset
IDO1	rs7820268	T	3.83E-03	0.2112	Brain putamen	129	Braineac
IDO1	rs7820268	T	1.77E-02	0.1259	Brain occipital cortex	129	Braineac
IDO1	rs7820268	T	3.61E-02	−0.1233	Brain cerebellar cortex	130	Braineac
IDO1	rs7820268	T	2.24E-02	−0.1385	Brain substantia nigra	101	Braineac
IDO1	rs7820268	T	4.50E-02	−0.1290	Brain thalamus	124	Braineac
IDO1	rs7820268	T	2.08E-02	−0.1831	Brain hippocampus	122	Braineac
IDO1	rs7820268	T	1.77E-83	>0 (*z* = 19.36)	Blood	2,116	BIOS QTL Browser
IDO1	rs7820268	T	1.59E-43	>0 (*z* = 13.83)	Blood	5,311	Blood eQTL browser
IDO1	rs7820268	T	7.31E-121	0.0900	Blood	5,257	FHS_eQTL

The rs7820268 position (hg19), 8_39777529_C_T (C>T); the threshold of significant association is 0.05; β > 0 and β < 0 means that this effect allele up-regulates and down-regulates gene expression, respectively; β = *z* × SE.

In summary, our findings could further support and expand the conclusions of previous studies. Moreover, in addition to blood, the immense influences of rs7820268 on IDO1 expression in six brain tissues were also detected, suggesting the promising functional studies of IDO1 in brain.
